# Chemoradiation for elderly patients (≥ 65 years) with esophageal cancer: a retrospective single-center analysis

**DOI:** 10.1186/s13014-022-02160-w

**Published:** 2022-11-17

**Authors:** Philipp Linde, Markus Mallmann, Anne Adams, Simone Wegen, Johannes Rosenbrock, Maike Trommer, Simone Marnitz, Christian Baues, Eren Celik

**Affiliations:** 1 Department of Radiation Oncology, Cyberknife and Radiation Therapy, Faculty of Medicine and University Hospital of Cologne, University of Cologne, Kerpener St 62, 50937 Cologne, Germany; 2 Center for Integrated Oncology (CIO), University Hospital of Cologne, Faculty of Medicine and University of Cologne, Kerpener St 62, 50937 Cologne, Germany; 3 Institute of Medical Statistics and Computational Biology, Faculty of Medicine and University of Cologne, Kerpener St 62, 50937 Cologne, Germany

**Keywords:** Carcinoma, Esophageal cancer, Chemoradiation, Elderly, Toxicity

## Abstract

**Background:**

Present studies on the efficacy and safety of curative chemoradiation therapy (CRT) with esophageal cancer reflect heterogenous results especially in elderly patients. The aim of this study was to evaluate the toxicity and efficacy of CRT in patients ≥ 65 years. In a cohort, the focus centered around treatment-related toxicity (CTCAE Grade > 3), overall survival as well as progression free survival, comparing these rates in-between patients older than 70 years to those younger than 70 years.

**Methods:**

A total of 67 patients older than 65 years (34 (50.7%) were older than 70 years) met the inclusion criteria for retrospective analysis (period from January 2013 to October 2017). Treatment consisted of radiotherapy and chemotherapy with carboplatin/paclitaxel or fluorouracil (5-FU)/cisplatin with the intention of neoadjuvant or definite chemoradiation. A sum of 67 patients received CRT (44 (65.6%) patients in neoadjuvant, 23 (34.4%) in definite intent). Of these, 22 and 12 patients were older than 70 years (50% and 52.2% in both treatment groups, respectively). Median age was 71 years and patients had a good physical performance status (ECOG 0: 57.6%, ECOG 1: 27.3%). Median follow-up was 24 months. Most patients had advanced tumour stages (T3 stage: n = 51, 79.7%) and nodal metastasis (N1 stage: n = 54, 88.5%). A subgroup comparison was conducted between patients aged ≤ 70 years and > 70 years.

**Results:**

In severe (CTCAE Grade 3–5) toxicities (acute and late), no significant differences were observed between both patient groups (< 70 years vs. > 70 years). 21% had acute grade 3 events, 4 patients (4%) had grade 4 events, and two patients (3%) had one grade 5 event. Late toxicity after CRT was grade 1 in 13 patients (22%), grade 2 in two (3%), grade 3 in two (3%), grade 4 in four (7%), and grade 5 in one (2%). Median overall survival (OS) of all patients was 30 months and median progression-free survival (PFS) was 16 months. No significant differences were seen for OS (32 months vs. 25 months; *p* = 0.632) and PFS (16 months vs. 12 months; *p* = 0.696) between older patients treated with curative intent and younger ones. Trimodal therapy significantly prolonged both OS and PFS (*p* = 0.005; *p* = 0.018), regardless of age.

**Conclusion:**

CRT in elderly patients (≥ 65 years) with esophageal cancer is feasible and effective. Numbers for acute and late toxicities can be compared to cohorts of younger patients (< 65 years) with EC who received the same therapies. Age at treatment initiation alone should not be the determining factor. Instead, functional status, risk of treatment-related morbidities, life expectancy and patient´s preferences should factor into the choice of therapy.

## Background

In 2020, 604,100 cases of esophageal cancer (EC) were newly diagnosed; the disease caused 544,076 deaths worldwide [1]. In Germany, EC makes up about 3.5 percent of all cancer deaths in men and 1.2 percent in women [2].

Squamous cell carcinomas (SCCs) represent the majority of histopathologic esophageal cancers in Western countries, but the incidence of adenocarcinomas (ACs) has increased significantly [3]. Treatment consisting of chemoradiation therapy (CRT) and surgery is considered the multimodal standard of care for patients with locally advanced EC [4–7].

Here, treatment of elderly EC patients can be challenging as they can present poor physiologic (performance) status as well as competing comorbidities. Treatment decisions should not only take into account patients’ age, but also their functional status, risk of treatment-related morbidities, life expectancy, and patients’ preference [8, 9]. Previously, a significant association between comorbidity, treatment tolerance and overall survival could be described based on a study of a series of 109 patients aged ≥ 70 treated with definite CRT (dCRT) [10]. Another retrospective single-center analysis showed that treatment of elderly patients with definite or neoadjuvant CRT can lead to significantly higher toxicity and far less favorable outcome [11]. The accessible evidence mirrors the general applicability of trial results to the elderly population, although often debarred—or at least underrepresented—in clinical trials [12].

To close this gap and examine CRT in elderly EC patients, we performed this retrospective single-center analysis from our institution investigating trimodal therapy and dCRT regimes in elderly patients with EC with a focus on acute and late toxicities, overall and progression free survival.

## Methods

We conducted a retrospective analysis of 67 patients aged ≥ 65 years with EC (cT1-4, any N, any M) who were treated either with neoadjuvant or definite CRT between January 2013 and October 2017 at our cancer center. Due to the time period covered by this study and practice-changing procedures, dose prescription, chemotherapy regimens and radiation techniques are different. Inclusion criteria were newly diagnosed histologically proven SCC or AC of the esophagus, patient age ≥ 65 years and neoadjuvant or definite CRT using conventional (anterior–posterior (APPA) fields, 3D-conformal multi-field, or intensity-modulated radiotherapy (IMRT)) techniques. Patients were excluded if they were treated for recurrent disease or if they had received prior CRT. We excluded incomplete records where radiotherapy was stopped prematurely and not applied up to the total prescribed dose. A systematic investigation of patients’ clinical charts and reports was performed in order to obtain patient and treatment characteristics, reported acute and late toxicities, and treatment-related outcomes.

### Chemoradiation

For radiotherapy planning, patients were simulated supine and immobilized with a universal/wing board for midthoracic to distal esophageal tumors or long mask for upper esophageal tumors. Contrast medium was administered both intravenously and orally, provided there were no contraindications (such as risk of aspiration or allergy). The gross tumour volume (GTV) was identified on the pre-chemotherapy extent of the disease, using the initial positron emission tomography in combination with a computed tomography (PET/) CT scan and endoscopy report. The entire esophageal wall, including any disease that extended through the wall, was contoured as GTV as well as any (PET/) CT-avid or enlarged lymph nodes. The clinical target volume (CTV) encompassed the peri-esophageal lymph nodes, mediastinal lymph nodes and the submucosal spread longitudinally along the esophagus. This required a 3–4 cm expansion on the GTV superiorly and inferiorly and a 1.0–1.5 cm radial expansion. The planning target volume (PTV) was generated adding 0.7 cm isotropically.

Radiotherapy was administered once a day, five times a week, except weekends and holidays, with a daily dose of 1.8 Gy. The total doses administered to PTV were 50,4 Gy and a sequential boost of 9 Gy to the GTV in dCRT and 41.4 Gy in nCRT, respectively.

Patients were assigned to chemotherapy (n = 65, 97%) according to a treatment plan based on performance status, comorbidity, and the presence of specific contraindications to the planned agents, which was developed by a multidisciplinary tumour board and finally prescribed by the treating radiation oncologist. Patients who were to receive taxane-based nCRT were planned for four administrations; five to six applications were targeted in the definitive setting. In 87% of patients, intravenous chemotherapy consisted of either cycles of carboplatin/paclitaxel (Carb/TAX; carboplatin [AUC 2 mg/mL per min] combined with paclitaxel [50 mg/m2 body-surface area], weekly, five times) or two courses of cisplatin/5-fluorouracil (CDDP/5FU; cisplatin [75 mg/m2 body-surface area] on the first day combined with 5-fluorouracil [1000 mg/m2] continuous infusion daily for four days).

The cisplatin-containing chemotherapy regimen was administered after adequate i.v. prehydration, manitol and i.v. antiemetics (5HT3 antagonists and dexamethasone), followed by i.v. posthydration. Taxane-based therapy was applied under premedication to prevent hypersensitivity reactions (dexamethasone, dimetinden maleate and H2 antagonists). Additional antiemetics (5HT3 antagonists, corticosteroids, dimenhydrinate and metoclopramide) were used at a patient's request to treat persistent nausea. During CRT, a complete blood count and serum chemistry test, including creatinine clearance were done once a week, more frequently if needed.

We considered dose reduction or treatment de-escalation of chemotherapy if grade 3 to 4 haematological toxicity occurred. An individual decision on dose reduction or discontinuation of chemotherapy was also made in case severe radiation-related toxicity occurred.

### Monitoring under radiochemotherapy and accompanying supportive measures

Acute treatment toxicity was assessed weekly during radiotherapy and daily during chemotherapy. If clinically indicated, toxicity was monitored more frequently. The documentation was standardized using the valid Common Terminology Criteria for Adverse Events (CTCAE) version in the respective period. Supportive care included management of pain, nausea or other side effects, nutritional counselling, enteral or parenteral nutrition, and supportive hospitalization if necessary.

### Follow-up

After CRT, all patients received follow-up appointments at our department, the first one 8–10 weeks after the end of treatment, followed by further appointments every three to six months afterwards during the first year. In addition, patients also received their oncological follow-up or surgery (when nCRT was performed) in the departments of medical oncology or visceral surgery. Radiooncological aftercare included medical history and clinical examination. We reviewed follow-up imaging (mostly computertomography and endoscopy results) of the tumour region (in most cases the scans were signed up by the medical oncologist) and scheduled them if they have not been performed yet. After the first year of follow-up, if no progressive or recurrent disease occurred, appointments were extended to every 6–12 months until disease progression or death; earlier if patients had complaints. Patients, medical oncologists, and their general practitioners were encouraged to report complications after CRT and were contacted if they missed a scheduled follow-up appointment. Recurrent disease was documented by histological biopsy if accessible. In case of missing information, the database of the University Hospital of Cologne was checked for information about survival or recurrence.

### Statistical analysis and ethical considerations

A person who is 65 years of age or older is often referred to as "elderly" [13, 14]. Three groups were created for analysis: Total collective, patients younger than 70 years, patients older than 70 years [15]. A subgroup comparison was conducted between patients aged ≤ 70 years and > 70 years. The latter were compared with each other. Survival data were estimated according to the Kaplan–Meier method [16]. OS was defined as the interval from the first day of treatment to death or to the last follow-up time point still alive. PFS was calculated from the first day of treatment until death or diagnosis of relapse (local or distant metastases) or last follow-up alive. Univariate analyses were performed using log rank testing and a Cox regression analysis [17]. A *p*-value of < 0.05 was defined as statistically significant. Acute and late toxicity was scored retrospectively according to CTCAE V5.0 [18]. Performance status was scored according to the ECOG index [19]. Calculations and data management were performed with SPSS®-statistics software v.26.0.0.1.

The study was conducted in accordance with the Declaration of Helsinki in its latest version. Due to the retrospective nature, from the point of view of the local ethics committee, there is no professional consultation obligation for the North Rhine physicians according to § 15 para. 1 of the professional code of conduct. All patients gave written informed consent before the start of treatment.

## Results

Median follow-up for the entire cohort was 24 months. Median age of the 67 patients in our cohort at the start of therapy was 71 years (range 65–82), 19 patients were female, 48 were male. 34 patients (50.7%) had SCC, 33 patients (49.3%) AC. The majority presented with an ECOG index between 0 and 1 (57.6% vs. 27.3%), T3 stage (n = 51; 79,7%) and N1 stage (n = 54; 88,5%). Most were treated with nCRT (n = 44; n = 22 > 70 years) and received surgery; 23 patients were treated with definite intent (n = 12 > 70 years). For detailed patient and treatment characteristics see Table 1.

**Table 1:** Patient and treatment characteristics (n)

*Sex*		*Grading*	
Female	19 (28.4%)	1	1
Male	48 (71.6%)	2	33
*Age*		3	25
Median	71 y	Unknown	8
Mean	71.6 y	*T-stage*	
Range	65–82 y	1	1
*ECOG*		2	10
0	38	3	51
1	18	4	2
2	6	Tx/Unknown	3
3	4	*N-stage*	
Unknown	1	0	3
*Tumor site*		1	54
Cervical	4	2	3
Upper thoracic	11	3	1
Middle thoracic	19	Nx/Unknown	6
Lower thoracic	33	*M-stage*	
*Histology*		0	63
Adeno	33	1	3
SCC	34	Mx/Unknown	1
*AJCC/UICC stage*^*I*^		*SCC*	*Adeno*
II		4	0
III		23	25
IVA		2	2
IVB		1	2
Unknown		4	4
*Chemotherapy*		*RT technique*	
Neoadjuvant	44	APPA (two-field)	11
Definite	23	3D-CRT (multi-field)	34
*Agents*		IMRT	19
Platinum-taxane-based	58	Unknown	3
Others	9		
*Charlson score*^*II*^		*SCC*	*Adeno*
≤ 1		27	25
> 1		7	8

*Adeno* adenocarcinoma, *IMRT* intensity modulated radiation therapy, *RT* radiation therapy, *SCC* squamous cell carcinoma, *3D-CRT* three-dimensional conformal radiation therapy, *Unknown* Due to the retrospective character of the study, *y* years

^I^8th edition AJCC/UICC staging of cancers of the esophagus and esophagogastric junction, ^II^The final score was calculated for each patient by considering all comorbid conditions present with the exclusion of EC

All patients completed radiotherapy with the RT dose we initially prescribed.

### Chemotherapy

58 of our 67 patients received concurrent platinum-taxan-based chemotherapy, four were treated with CDDP/5FU, two patients received taxol and one carboplatin monotherapy. Two patients did not receive any chemotherapy based on the interdisciplinary assessment of the treatment team.

### Treatment-related toxicity

#### Acute

Severe adverse events (CTCAE Grade 3–5 acute toxicity) were found in 20 cases; nine in the group older than 70 years. More specifically, 14 patients (21%) had grade 3 events, 4 patients (4%) had grade 4 events and two patients (3%) had one grade 5 event. Most reported grad 3 and 4 events were (odyno-)dysphagia, nausea, and fatigue. Patients with grade 5 toxicity presented as follows: 80 years, female, ECOG 3, CCI 2, cT3, SCC, and dCRT (taxol mono) and 68 years, male, ECOG 2, CCI 2, cT4, SCC, and dCRT (CDDP/5FU), respectively. Grade 5 toxicity in both patients was related to haematotoxicity. In detail, there were no significant differences in treatment-related acute toxicity between the groups younger vs. older than 70 years.

#### Late

Grade 3 late toxicity (or higher) was found in seven of 58 evaluable cases; two in the group older than 70 years. In general, maximum late toxicity after CRT was grade 1 in 13 patients (22%), grade 2 in two (3%), grade 3 in two (3%), grade 4 in four (7%), and grade 5 in one (2%); mainly (odyno-)dysphagia. The patient with grade 5 toxicity presented as follows: 67 years, male, ECOG 2, CCI 5, cT3, SCC, and dCRT (Carb/TAX). Grade 5 toxicity was related to fistula. Again, there were no significant differences in treatment-related late toxicity between the groups younger vs. older 70 years. For detailed analysis of acute and late toxicity see Tables 2 and 3.

**Table 2:** Acute treatment related toxicities according to CTCAE v5.0 for both patient groups, absolute number of patients (n = 67)

CTC grade	0				1				2				3				4				5			
	$\le$ 70 y	%	$>$ 70 y	%	$\le$ 70 y	%	$>$ 70 y	%	$\le$ 70 y	%	$>$ 70 y	%	$\le$ 70 y	%	$>$ 70 y	%	$\le$ 70 y	%	$>$ 70 y	%	$\le$ 70 y	%	$>$ 70 y	%
Hematological	3	9.1	7	20.6	9	27.3	10	29.4	15	45.5	13	38.2	3	9.1	2	5.9	2	6.1	1	2.9	1	3.0	1	2.9
Odynodysphagia	20	60.6	19	55.9	3	9.1	10	29.4	3	9.1	2	5.9	6	18.2	3	8.8	1	3.0	0	0	0	0	0	0
Nausea	30	90.9	28	82.4	2	6.1	6	17.6	1	3.0	0	0	0	0	0	0	0	0	0	0	0	0	0	0
Skin toxicity	21	63.6	22	64.7	10	30.3	11	32.4	2	6.1	0	0	0	0	1	2.9	0	0	0	0	0	0	0	0
Fatigue	30	90.9	24	70.6	2	6.1	7	20.6	1	3.0	2	5.9	0	0	1	2.9	0	0	0	0	0	0	0	0
Cardiopulmonary	32	97.0	34	100.0	0	0	0	0	0	0	0	0	0	0	0	0	1	3.0	0	0	0	0	0	0
Inappetence/ weight loss	32	97.0	32	94.1	1	3.0	1	2.9	0	0	0	0	0	0	1	2.9	0	0	0	0	0	0	0	0
Other	29	87.9	30	88.2	2	6.1	3	8.8	2	6.1	0	0	0	0	1	2.9	0	0	0	0	0	0	0	0

*y* years

**Table 3:** Late treatment related toxicities according to CTCAE v5.0 for both patient groups, absolute number of patients (n = 58)

	All grades		Grade 3–5			
	n	n	n	%	n	%
	$\le$ 70 y	$>$ 70 y	$\le$ 70 y		$>$ 70 y	
Odynodysphagia	28	30	5	17.8	2	6.6
Nausea	28	30	0	0	0	0
Skin toxicity	28	30	0	0	0	0
Fatigue	28	30	0	0	0	0
Cardiopulmonary	28	30	0	0	0	0
Inappetence/ weight loss	28	30	0	0	0	0
Fistula	28	30	1	3.6	0	0
Other	27	30	0	0	0	0

*y* years

#### Survival analysis

**Fig. 1:**
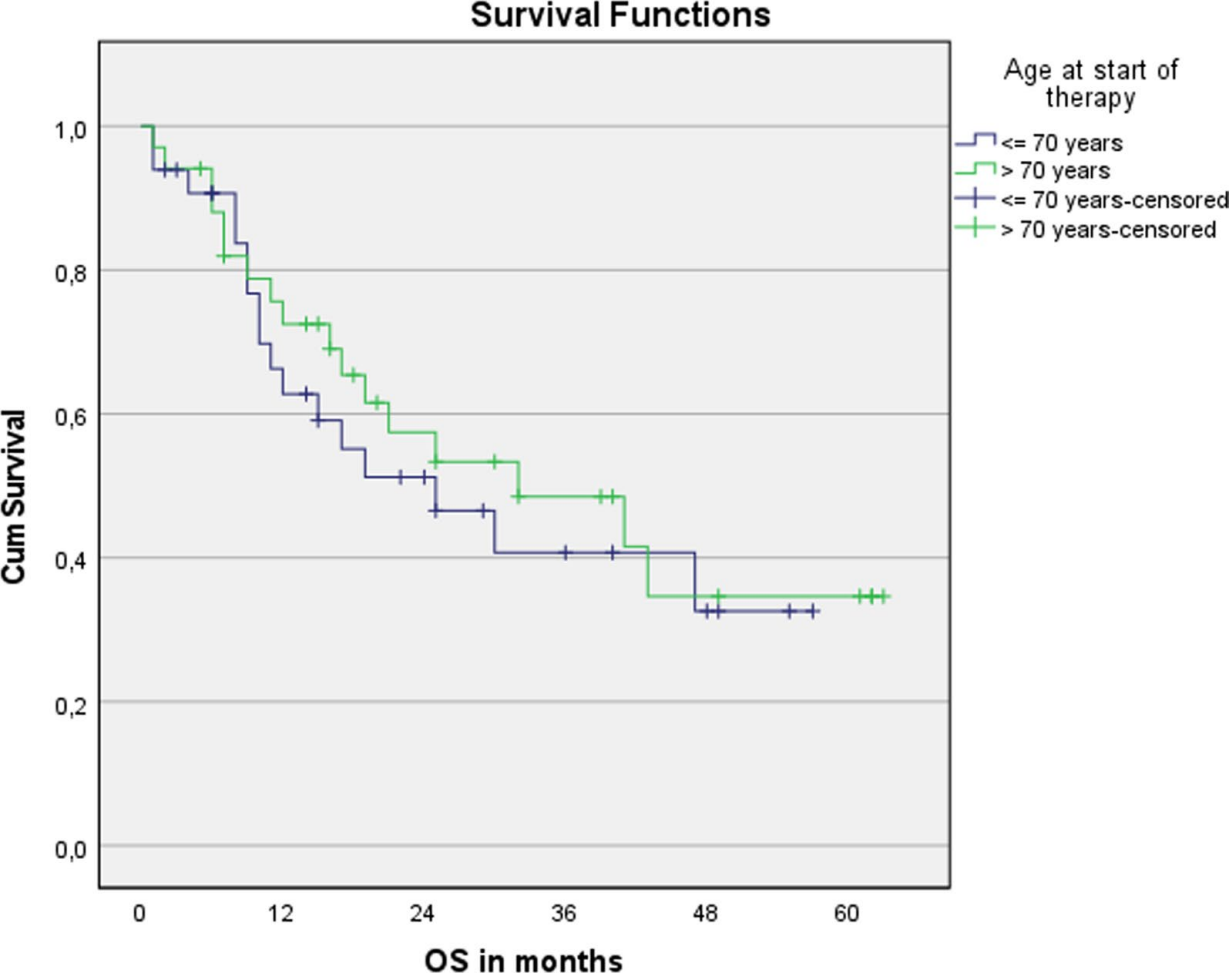
Overall survival for patients younger or older than 70 years. n = 67. Log Rank (Mantel-Cox): *p* = 0.632

**Fig. 2:**
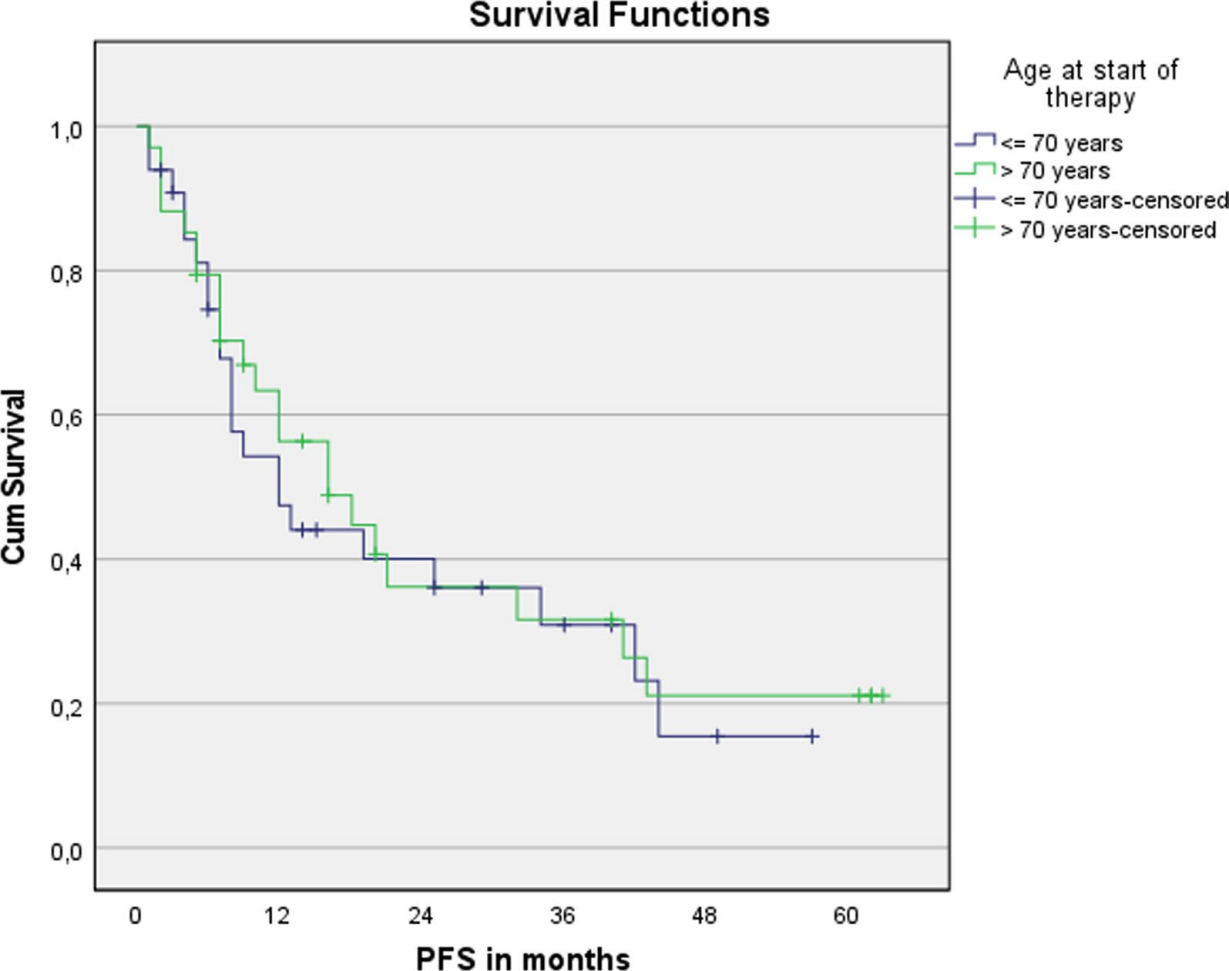
Progression free survival for patients younger or older than 70 years. n = 67. Log Rank (Mantel-Cox): *p* = 0.696

**Fig. 3:**
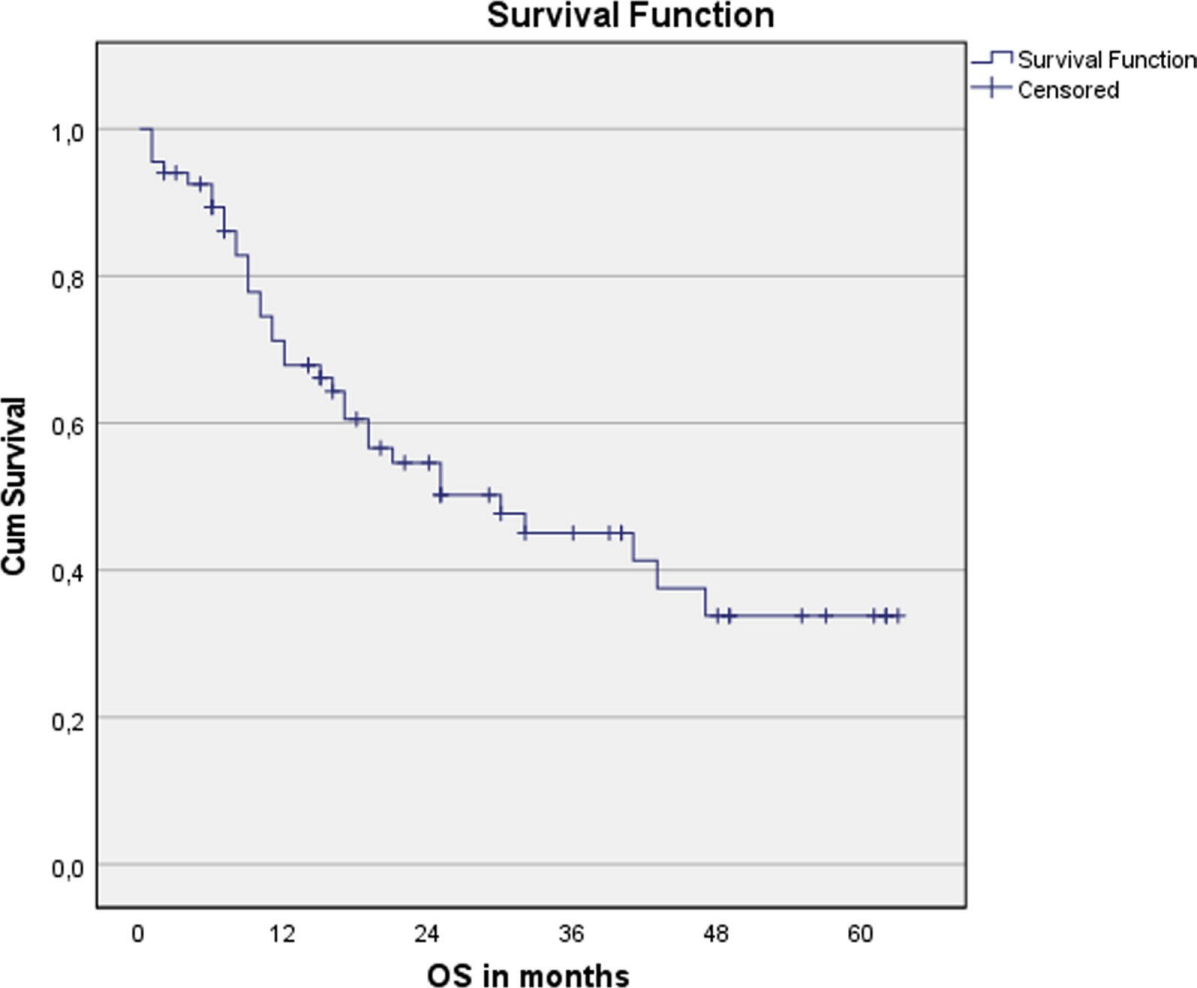
Overall survival for the entire cohort. n = 67

**Fig. 4:**
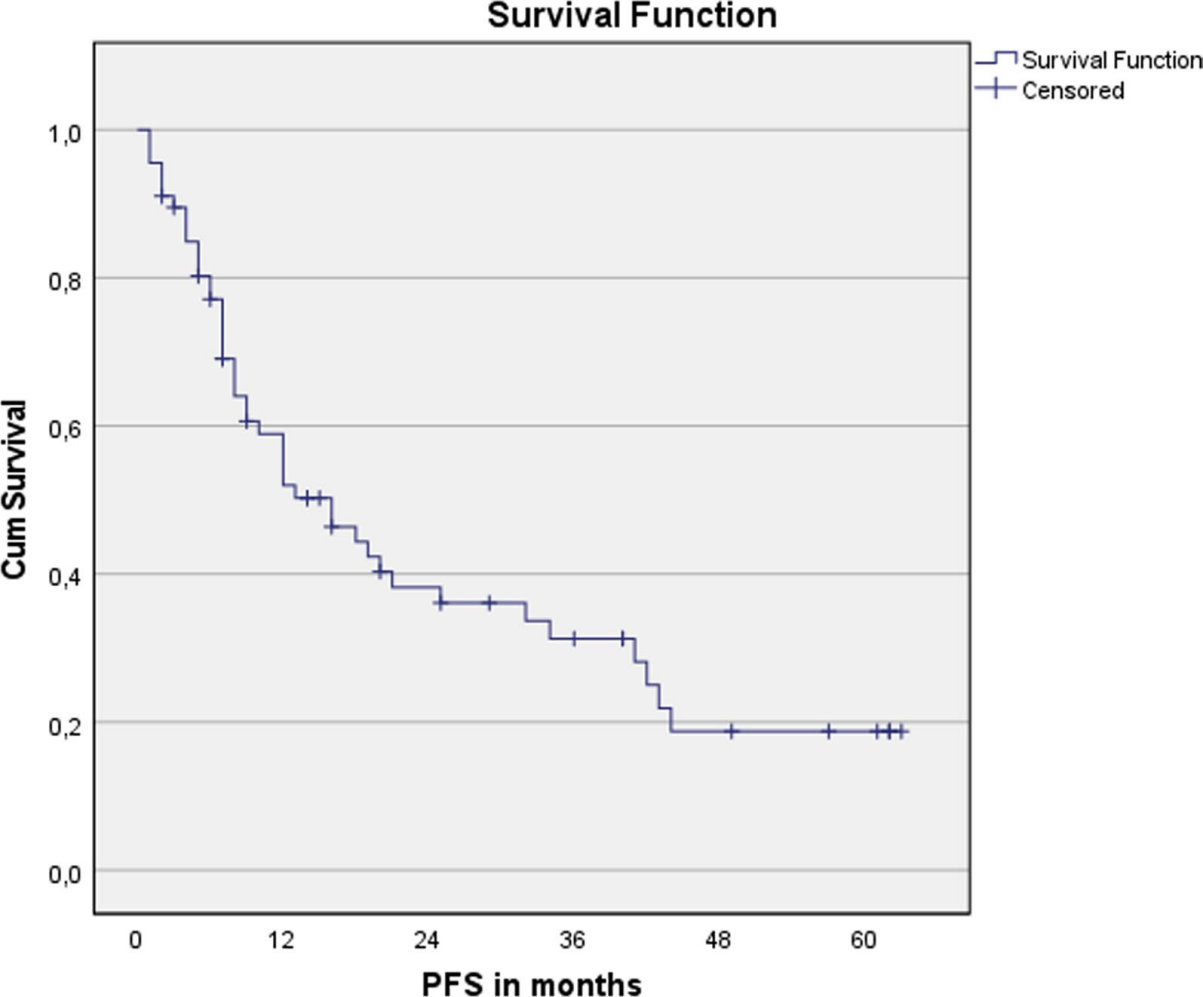
Progression-free survival for the entire cohort. n = 67

No significant differences were seen for median overall survival (32 months vs. 25 months; *p* = 0.632) and progression-free survival (16 months vs. 12 months; *p* = 0.696) between older patients (> 70 years) treated with curative intent and younger ones (< = 70 years), see Figs. 1 and 2, respectively. The median OS of all patients ≥ 65 years was 30 months and the median PFS was 16 months, see Figs. 3 and 4, respectively. The median OS for SCC patients was 19 months vs. 32 months for AC (*p* = 0.679).

**Fig. 5:**
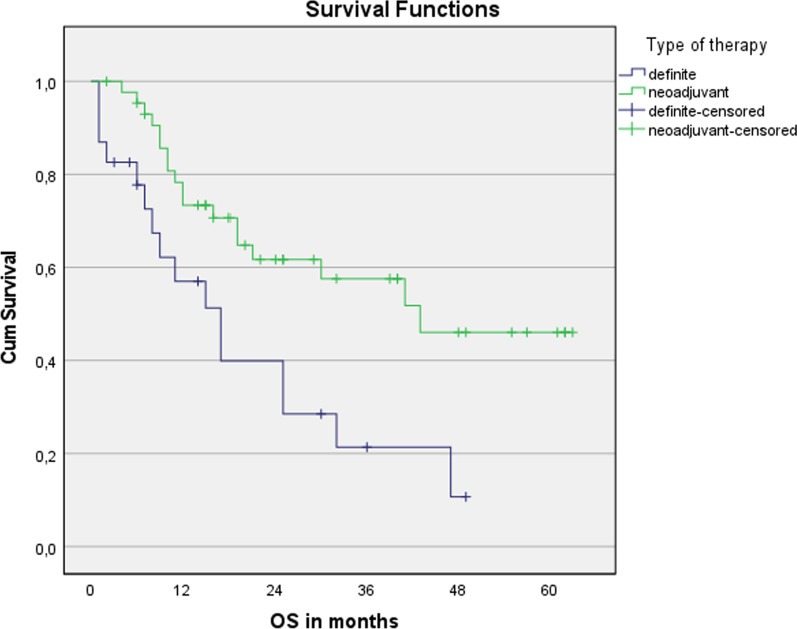
Overall survival for nCRT and dCRT. n = 67. Log Rank (Mantel-Cox): *p* = 0.005

**Fig. 6:**
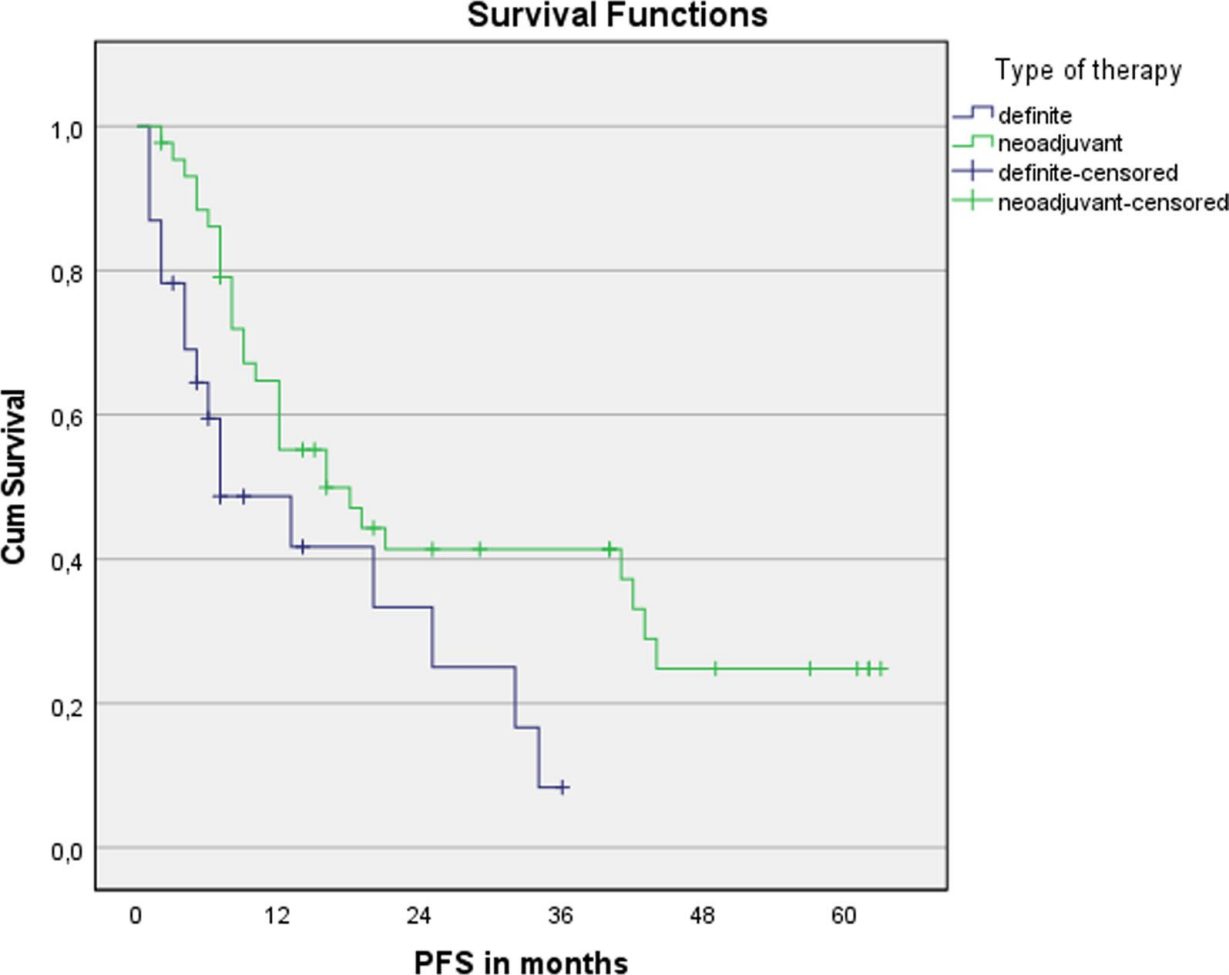
Overall survival for nCRT and dCRT. n = 67. Log Rank (Mantel-Cox): *p* = 0.018

nCRT showed significantly advantages in OS and PFS compared to dCRT (43 months vs. 17 months; *p* = 0.005; 16 months vs. 7 months; *p* = 0.018), see Figs. 5 and 6, respectively. In the univariate Cox regression model, nCRT significantly affected OS and PFS (*p* = 0.008; *p* = 0.023), too. The trend shown is independent of age. ECOG index 0–1 was one parameter significantly affecting both OS and PFS (*p* = 0.001; *p* = 0.003); G3-staged carcinomas showed significantly improved PFS (*p* = 0.044) for patients <  = 70 years.

Detailed results of univariate Cox regression analysis are demonstrated in Table 4.

**Table 4:** Univariate Cox regression analysis. HR > 1 indicates a greater risk for the group > 70y; HR < 1 indicates a smaller risk for the group > 70y

Parameter	OS HR [95% CI]	*p*-value	PFS HR [95% CI]	*p*-value
Treatment Regimen (nCRT vs. dRCT)	0.399 [0.203; 0.785]	0.008	0.477 [0.252; 0.902]	0.023
Age > 70 y vs. $\le$ 70 y)	0.850 [0.433; 1.666]	0.636	0.891 [0.493; 1.610]	0.701
Sex				
(Female > 70 y vs. $\le$ 70 y)	0.782 [0.205; 2.982]	0.719	0.605 [0.168; 2.173]	0.441
(Male > 70 y vs. $\le$ 70 y)	0.773 [0.340; 1.759]	0.539	1.032 [0.511; 2.083]	0.931
ECOG (2–3 vs. 0–1)	3.825 [1.738; 8.416]	< 0.001	3.096 [1.460; 6.565]	0.003
T-stage (> 70 y vs. $\le$ 70 y)				
T3	0.958 [0.429; 2.140]	0.917	0.973 [0.496; 1.908]	0.936
Grading (> 70 y vs. $\le$ 70 y)				
G2	0.379 [0.133; 1.081]	0.070	0.487 [0.204; 1.164]	0.105
G3	2.328 [0.819; 6.619]	0.113	2.585 [1.025; 6.520]	0.044
Histology ($>$ 70 y vs. $\le$ 70 y)				
SCC	0.462 [0.174; 1.227]	0.121	0.454 [0.172; 1.193]	0.109
AC	1.968 [0.625; 6.197]	0.248	1.495 [0.632; 3.536]	0.360

*HR *Hazard ratio, *CI *Confidence interval, *OS *Overall survival, *PFS *Progression-free survival, *y* years

## Discussion

With the overall increase in average life expectancy, the number of elderly EC patients is increasing year by year. In this study, we evaluated the toxicities and the outcome of nCRT and dCRT for EC in elderly patients younger than 70 years (minimum was 65 years of age) compared with patients out of the same cohort but older than 70 years.

30 years ago, the usefulness and compatibility of definite concurrent chemoradiation and its advantages in terms of survival compared to radiation alone were already demonstrated in randomized trials [20]. In comparison to surgery alone, nCRT significantly improved the survival rate of patients with curative therapy approach for esophageal or GEJ carcinoma by further developments in the last decade [6].

Unfortunately, elderly patients were underrepresented in the CROSS trial. The median patient age in the original study was 60 years. Several data suggest that trimodal therapy for the elderly is feasible, but without impact on survival benefit for patients over 70 years [21, 22]. Maybe this finding should be carefully considered, as the benefit of neoadjuvant therapy for patients with advanced T or N category is well established [23, 30].

Nevertheless, elderly patients who formally meet the criteria for neoadjuvant treatment followed by esophagectomy should be considered for nCRT depending on their individual comorbidities [24, 25, 27].

Our data show that curative CRT of patients with any EC older than 70 years result in no significant differences regarding acute and late toxicities as in patients younger than 70 years. The results of Lu et al. indicated that the treatment of patients with EC aged ≥ 75 years with CRT was effective and an age of 75 years did not affect the frequency of adverse events [26]. A pooled analysis of three clinical trials could show that patients > or = 70 years with advanced EC benefitted from the addition of platinum-based chemotherapy with respect to tumour regression, symptomatic response, and survival, but without increased acute or late toxicities, too [27]. Song et al. analyzed elderly patients (age ≥ 70 years) treated with platinum-taxane-based CRT resulting in tolerable toxicities, too [28]. Grade ≥ 3 leukopenia was observed, and the most common nonhematologic toxicity was esophagitis with grade 3 and 4 toxicities. Also Zhao et al. presented data of 86 patients ≥ 70 years receiving single- or double-agent concurrent CRT with slightly higher acute toxicities in the double-agent cohort (grade ≥ 2 neutropenia and gastrointestinal reactions) [29]. Our data agree that patients with SCC, proximal tumor location, and age less than 70 years are at increased risk for fistulae during or after chemoradiation [30]. This group of patients may benefit from more intensive therapy monitoring, follow-up, and radiomics [31].

The retrospective comparisons of Münch et al. [32, 33] between the two common chemotherapy regimes (CDDP/5FU or Carb/TAX) underline our results in relation to the low moderate acute and late toxicities due to chemotherapy in our cohort of elderly patients. Carb/TAX is considered more tolerable than platinum-fluoropyrimidine [34–36].

However, our data reveal that the risk of significant myelotoxicity does not increase with patients’ age [28]. Remarkably, in a large-scale study of the CROSS regimen in patients over 75 years of age, longer OS was observed after nCRT followed by surgery compared with surgery alone or dRCT, with no differences in postoperative mortality [37]. In contrast, Haefner and Minsky et al. found no difference in outcomes between cohorts receiving nCRT followed by surgery and dCRT stating that age and comorbidities should not be evaluated alone for the decision for dCRT versus nCRT followed by surgery [38, 39].

Qiu et al. could show that in a cohort of 855 elderly EC patients, the prognosis of elderly patients treated with chemotherapy was better than that of treatment without, regardless of whether the patients were treated with surgery or radiotherapy, including comparable toxicities [40]. Mantziari et al. pointed out that fewer elderly patients are being offered nCRT followed by surgery compared to younger ones, even though the histological response is at least as good as in younger patients. Not surprisingly, after trimodal therapy, complications (cardiovascular > pulmonal) are more common in older patients [41]. Here, patient selection should be performed carefully. nCRT followed by surgery may offer a survival benefit for elderly patients with incomplete clinical response to treatment [42].

Age per se should not be the sole factor for or against trimodal therapy [43–46]. Scoring systems like P-POSSUM or O-POSSUM and predictive models as published by Steyerberg et al. and, more recently, by the International Esodata Study group should be considered by stratifying our patients for more or less aggressive therapy regimens [47–50].

Patient-reported outcome measurement, quality of life (QOL) scoring and individual co-morbidities and the known pharmacokinetic properties and modes of action in the elderly must be also taken into consideration when stratifying the different modalities of therapy for elderly patients with EC [51–56].

More than half of our patient cohort were treated with 3D-CRT. Münch and Haefner compared 3D-CRT and modern radiation techniques such as IMRT for nCRT followed by surgery or dCRT in patients with esophageal cancer [37, 57]. Interestingly, no significant differences were found in terms of PFS and OS; still it is important to note that the use of modern radiotherapy techniques in patients undergoing nCRT was associated with a lower dose to organs at risk [37].

Of note, our retrospective analysis had some limitations. Treatment adherence is one of the most important endpoints in the treatment of frail or elderly patients. It should be recorded after all patients who have started chemoradiation with curative intent have been registered. Unfortunately, due to the retrospective nature of the study, we are not able to capture the total number of the collective, which we have to cite as a major limitation. It is therefore clear that our results should be interpreted with caution. Secondly, we were unable to adjust for multiple potential confounders and detect differences in this study. Finally, each elderly patient had an individual comorbidity and there was a lack of information regarding comorbidities, which may have introduced bias in the assessment of the benefit of therapy.

Nevertheless, our results underline both the feasibility and the need for modified inclusion concepts in older esophageal cancer patients. Further large prospective studies or randomized trials are still needed in order to validate the optimal modification strategy for CRT and establishment for age-based standards in elderly EC patients. Studies should consider imperative factors such as a patient's life expectancy, comorbidity, and a geriatric assessment. Wishes and expectations of the individual patient should also be included in the decision-making process. Key criteria should be post-therapy mortality and morbidity rates, rates of acute and late side effects of (C)RT, and short- and long-term effects in regard of QOL.

## Conclusion

Chemoradiation offers a feasible therapy for elderly patients. Curative CRT of patients with any EC older than 70 years results in comparable rates of acute and late toxicities as in patients < 70 years. Age at treatment initiation alone should not be a determining factor alone, rather an individual’s functional status, risk of treatment-related morbidities, life expectancy and their preference should be included. With improvements in tumour staging and treatment options, similar outcomes to younger cohorts appear to be achievable.

## Data Availability

The present data are summarized in this paper. The complete dataset can be retrieved from the authors upon formal request from interested readers.

## Abbreviations

3D-CRT: 3-Dimensional conformal radiotherapy
AC: Adenocarcinoma
Carb/TAX: Carboplatin and paclitaxel
CDDP/5FU: Cisplatin and 5-fluoruracil
dCRT: Definite chemoradiation therapy
EC: Esophageal cancer
GEJ: Esophagogastric-junction
GTV: Gross target volume
Gy: Gray
IMRT: Intensity-modulated radiotherapy
nCRT: Neoadjuvant chemoradiation therapy
OS: Overall survival
PET/CT: Positron emission tomography/computed tomography
PFS: Progression-free survival
PTV: Planning target volume
SCC: Squamous cell carcinoma
QOL: Quality of life
